# Effects of aging on calcium channels in skeletal muscle

**DOI:** 10.3389/fmolb.2025.1558456

**Published:** 2025-03-19

**Authors:** Mingyi Dong, Andrés Daniel Maturana

**Affiliations:** Department of Applied Biosciences, Graduate School of Bioagricultural Science, Nagoya University, Nagoya, Japan

**Keywords:** calcium, skeletal muscle, aging, ion channels, sarcopenia

## Abstract

In skeletal muscle, calcium is not only essential to stimulate and sustain their contractions but also for muscle embryogenesis, regeneration, energy production in mitochondria, and fusion. Different ion channels contribute to achieving the various functions of calcium in skeletal muscles. Muscle contraction is initiated by releasing calcium from the sarcoplasmic reticulum through the ryanodine receptor channels gated mechanically by four dihydropyridine receptors of T-tubules. The calcium influx through store-operated calcium channels sustains the contraction and stimulates muscle regeneration. Mitochondrial calcium uniporter allows the calcium entry into mitochondria to stimulate oxidative phosphorylation. Aging alters the expression and activity of these different calcium channels, resulting in a reduction of skeletal muscle force generation and regeneration capacity. Regular physical training and bioactive molecules from nutrients can prevent the effects of aging on calcium channels. This review focuses on the current knowledge of the effects of aging on skeletal muscles’ calcium channels.

## Introduction

Skeletal muscles’ work produces movements and actions in vertebrates. Over 600 human skeletal muscles represent about 40% of human body mass. Skeletal muscles are divided into two major groups: slow-twitch type I and fast-twitch type II (Frontera and Ochala, 2015). Skeletal muscles convert chemical energy into mechanical force to support the body, drive movements, and safeguard bones and organs. Skeletal muscles are formed by an assembly of muscle fibers generated by the fusion of myoblasts in a process called myogenesis (Luo et al., 2021). The muscle fibers are bundles of myofibrils assembled in repetitive structures called sarcomeres. Sarcomeres are contractile units limited on each side by the Z-discs made of α-actinin that anchor protein filaments made of actin, myosin, and titin filaments (Frontera and Ochala, 2015).

Myosin and actin filaments can physically interact when calcium (Ca^2+^) ions bind to troponin, provoking the displacement of tropomyosin-troponin on actin, allowing myosin motor protein to bind and crawl on actin filament, shortening the sarcomere length, and thus contracting the muscle (Brunello and Fusi, 2024). Ca^2+^ ions are released from the sarcoplasmic reticulum (SR) following excitation-contraction coupling (ECC) activation (Bolaños and Calderón, 2022). Ca^2+^ ion is a key player in skeletal muscle activation and the sustainability of contraction and energy production by mitochondria, myogenesis, and muscle regeneration (Sinha et al., 2022; Gherardi et al., 2025).

In the plasma membrane (sarcolemma), SR, or mitochondria membranes, various ion channels form selective pathways for Ca^2+^ to flow along its electrochemical gradient into the cytosol or in mitochondria to accomplish its functions (Table 1). Ca^2+^ is released from the SR through Ryanodine Receptor type 1 (RYR1) channels. A single RYR1 channel connects with four L-type voltage-gated Ca^2+^ channels, called dihydropyridine receptors (DHRP) in skeletal muscles, of the T-tube membrane, forming a tetrad. DHPRs sense the sarcolemma depolarization initiated at the neuromuscular junction and mechanically gate the RYR1 channel to release Ca^2+^ from the SR, initiating muscle contraction (Pearce et al., 2023). In addition to its key role in triggering and sustaining muscle contraction, Ca^2+^ ions influx through the store-operated Ca^2+^ channels, Orai1 and Transient Receptors Potential Canonical, and the mechanically gated ion channel, Piezo1, are essential for myogenesis. Myogenesis occurs both during embryogenic development and muscle regeneration following similar stages: differentiation of muscle progenitor cells (satellite cells) into myoblasts and then myocytes. Myocytes fuse to form multinucleated myotubes. Myotubes then mature into myofibers. Myocytes can also directly fuse with already-formed myofibers. Ca^2+^ signals are essential in different stages of myogenesis (Sinha et al., 2022; Tsuchiya et al., 2018). In mitochondria, Ca^2+^ is essential to stimulate the tricarboxylic acid cycle to produce ATP (adenosine triphosphate), the energy molecule necessary for muscle contraction (Gherardi et al., 2025).

**TABLE 1:** Skeletal Muscles Calcium Channles.

Channel name	Pore gene	Pore protein	Accessory protein	Gating mechanism	Cellular localization	Aging effects
DHRP	*CACNA1S*	CaV1.1 (α1S)	*ß1*, α2δ,γ, STAC3	Membrane depolarization	T-tubules	Expression↓, ECC uncoupling
RYR1	*RYR1*	RYR1	FKBP12(Calstabin 1)	Mechanical with ECC, Ca^2+^ binding	SR terminal cisternae	Leaky channel, ECC uncoupling
IP3R	*ITPR1*	IP3R1	None Identified	IP3, Ca^2+^ binding, ATP modulation	SR	Expression ↓
TRPC1	*TRPC1*	TRPC1	None Identified	SR Ca^2+^ depletion STIM1 oligomerization, mechanical stress	Plasma Membrane	Expression ↓
TRPC3	*TRPC3*	TRPC3	None Identified	SR Ca^2+^ depletion STIM1 oligomerization	Plasma Membrane	?
TRPC4	*TRPC4*	TRPC4	None Identified	SR Ca^2+^ depletion STIM1 oligomerization	Plasma Membrane	?
ORAI1	*ORAI1*	ORAI1	None Identified	SR Ca^2+^ depletion STIM1 oligomerization	Plasma Membrane	Expression ↓
Piezo1	*PIEZO1*	PIEZO-1	None Identify	Mechanical force, membrane potential	Plasma Membrane	?
MCU	*MCU, MCUb, EMRE*	MCU, MCUb, EMRE	MICU1, MICU2, MICU3	Ca^2+^-dependent	Mitochondria	Expression ↓

The table summarizes skeletal muscles Ca^2+^ channels’ gene, protein name, accessory proteins name, gating mechanisms, cellular location in myofibers, and aging effects (SR-sarcoplasmic reticulum, ECC-excitation contraction coupling, ↓-decrease).

Skeletal muscle aging is a progressive loss of muscle mass and force generation, with onset in humans at around 40–50 years. Muscle aging is a slow and steady process of about 1%–4% annual mass loss. A condition with a higher muscle mass loss rate than the average decrease is called sarcopenia (Wall et al., 2013).

Regular physical exercise and nutrition attenuate the effects of skeletal muscle aging (McKendry et al., 2024; Yadav et al., 2022). At the cellular level, muscle aging is marked by genomic instability, telomer shortening, epigenetic alteration, inflammation, increased mitochondrial instability, cellular senescence, increased autophagy, loss of neuromuscular junction, and disruption of Ca^2+^ homeostasis and signaling (Granic et al., 2023). The aging-dependent disruption of Ca^2+^ homeostasis and signaling in skeletal muscles arises through the alteration of Ca^2+^ channels’ activity and expression (Table 1). Here, we review how aging affects the various skeletal muscles’ Ca^2+^ channels, resulting in the reduction of the excitation-contraction coupling efficiency, muscle contraction, energy production, and regeneration.

### L-type voltage-gated Ca^2+^ channels in aging skeletal muscle

Voltage-gated Ca^2+^ channels (VGCCs) are Ca^2+^-selective channels whose gating is controlled by the membrane depolarization following an action potential. Electrophysiologically, VGCCs can be divided into two groups: those activated by weak membrane depolarization, called LVA, for low voltage activation, and those activated by strong membrane depolarization, called HVA, for high voltage activation. Among the LVA-VGCCs are the T-type VGCCs, characterized by a small transient Ca^2+^ ion conductance with fast inactivation. T-type VGCCs are encoded by three distinct genes: *CACNA1G*, *CACNA1H*, and *CACNA1I*. These three genes encode for the pore subunit of T-channels, CaV3.1 (α1G), CaV3.2 (α1H), and CaV3.3 (α1I), respectively (Catterall, 2011; Catterall et al., 2020). HVA-VGCCs are the L-, P/Q-, N-, and R-types VGCCs characterized by larger Ca^2+^ conductance, slow inactivation, and specific pharmacology. P/Q-type, N-type, and R-type are neuronal VGGCs, and their channels are formed respectively by the pore subunit CaV2.1 (α1A), CaV2.2 (α1B), and CaV2.3 (α1E), encoded by three distinct genes, *CACNA1A*, *CACNA1B*, and *CACNA1E*. The pore subunit of L-type VGCCs, CaV1.1 (α1S), CaV1.2 (α1C), CaV1.3 (α1D) and CaV1.4 (α1F) are encoded by four distinct genes, *CACNA1S*, *CACNA1C*, *CACNA1D*, and *CACNA1F*. The pore subunit of HVA-VGCCs is associated with three other subunits named, β, α2δ, and γ, also encoded by distinct different genes *CACNB1* to *4*, *CACNA2D1* to *4*, and *CACNG1* to *eight* respectively (Catterall et al., 2020). These subunits are necessary for the plasma membrane localization of the pore subunit and the modulation of the channel’s activity (Yao et al., 2024).

### L-type voltage-gated Ca^2+^ channels’ structure and function

**FIGURE 1:**
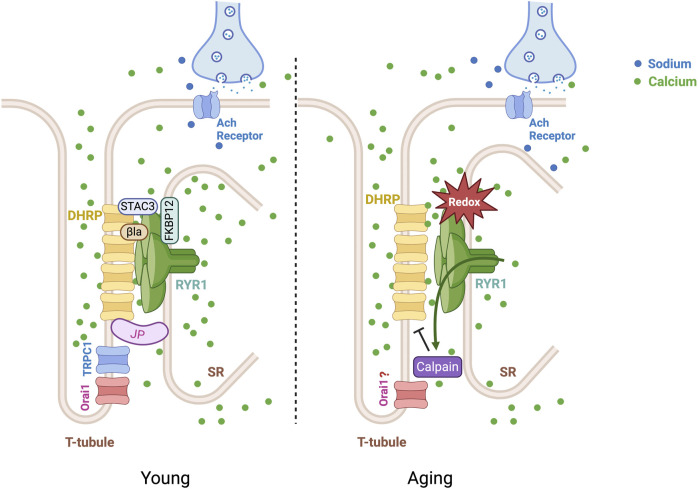
Ca^2+^ channels in skeletal muscle contraction. Four dihydropyridine receptors (DHRP) at the T-tubules form a triad with a single RYR1 on the sarcoplasmic reticulum (SR) membrane. Triad junctions are linked by Junctophilins (JPs), connecting the T-tubule membrane to the SR membrane. RYR1 and DHRP interact with the assistance of β1a and STAC3. In muscle contraction, DHRPs transduce the membrane depolarization triggered by the action potential into a mechanical gating of RYR1, triggering the release of SR-stored Ca^2+^ ions. The store-operated calcium channels, Orai1 and Transient Receptors Potential Canonical (TRPC), upon the SR Ca^2+^ depletion, initiate an extracellular Ca^2+^ influx to refill the SR with Ca^2+^ and sustain the contraction. Under oxidative stress and aging, the RYR1 becomes leaky, constantly releasing Ca^2+^. The leak of Ca^2+^ activates the Ca^2+^-activated protease calpain in the cytosol, impairing the Ca^2+^ release unit through protein degradation.

L-type VGCC of skeletal muscles, originally called the dihydropyridine receptor (DHRP), are formed by the pore subunit α1S (CaV1.1) associated with the cytoplasmic β1a subunit, the two membrane subunits α2δ1, and γ1. The complete structure of the skeletal DHRP, α1S (CaV1.1), associated with β1a, α2δ1, and γ1 has been solved (Wu et al., 2015). The pore subunit CaV1.1 is a large protein formed by a single peptide with four domains, containing each six transmembrane α-helix called S1 to S6. The pore is formed by the S5 and S6 helixes of each domain, and the loop between both helixes forms the Ca^2+^selective filter. The S4 helix of each domain functions as a voltage sensor, and their movement upon a membrane potential variation triggers the opening of the channel’s pore, letting Ca^2+^ ions flow into the cell along the concentration electrochemical gradient. The movement of the voltage sensor, S4 helix, of the CaV1.1 domain III, was recently demonstrated to gate the RYR1 upon sarcolemma depolarization (Pelizzari et al., 2024). In muscle fibers, DHRP channels are located in T-tubules, and four channels form a triad that is physically linked to a single RYR1 on the membrane of the sarcoplasmic reticulum (Figure 1). The Ca^2+^ ions influx through the DHPR are not required for skeletal muscle contraction. The major function of the DHRP is to transduce the membrane depolarization triggered by the action potential into a mechanical movement of the voltage sensor. DHPRs will, in turn, mechanically gate the RYR1, triggering the release of Ca^2+^ ions stored in the SR to stimulate muscle contraction. The role of the DHRP Ca^2+^ influx function is still not fully understood. The DHRP-dependent Ca^2+^ signal could play a role in the regulation of immediate early gene expression (Araya et al., 2003).

Voltage-gated Ca^2+^ channel subunits encoding genes are subject to alternative splicing, generating various isoforms with different electrophysiological properties (Lipscombe et al., 2013). In skeletal muscles, the pore subunit CaV1.1 has only two known splice variants. The CACNA1S gene is composed of 44 exons. Exon 29 is subject to alternative splicing. In the embryo, exon 29 is skipped, generating a shorter splice variant called CaV1.1e. CaV1.1e shows an increased Ca^2+^ current compared to the adult splice variants, CaV1.1a, that include exon 29 (Tuluc et al., 2009). The skipping of exon 29 drastically modified the L-type Ca^2+^ channel’s electrophysiological properties of CaV1.1e compared to CaV1.1a with a shifted activation to more negative potentials. However, interestingly, the reduced current observed depended on the interaction of the γ1 subunit, suggesting an inhibitory interaction between γ1 and exon 29.

### L-type voltage-gated Ca^2+^ channels’ role in aging

Aging skeletal muscles, particularly in fast type II muscle fibers, have an altered excitation-contraction coupling caused by a decreased coupling, called uncoupling, between CaV1.1 and RYR1 (Figure 1) (Wang et al., 2000; Renganathan et al., 1997) This uncoupling is caused by a decreased expression of CAV1.1 in T-tubules (Delbono et al., 1995). With less CaV1.1 located in T-tubules, the formation of complete CaV1.1 tetrads coupled to one RYR1 channel is reduced in aged myotubes derived from human satellite cells from old patients compared to young patients (Pietrangelo et al., 2024). Aging comes with a decreased expression of IGF-1 (Insulin Growth Factor-1) (Ascenzi et al., 2019). IGF-1 has been shown to stimulate the transcription of CaV1.1 upon activation of the CREB (cAMP response element binding protein) transcription factor (Zheng et al., 2002). An age-dependent reduction of IGF-1 stimulation could thus explain the reduced expression of CAV1.1 expression, leading to a reduced number of DHRP in T-tubules. Interestingly, another transcriptional regulator of CaV1.1 was also identified to play a role in the aged-dependent reduction of CaV1.1, Troponin T3. Troponin T3 (TnT-T3) is an isoform of Tropinin, essential for Ca^2+^-dependent muscle contraction. TnT-T3 is located both in the cytosol and nucleus. TnT-T3 was found to stimulate the transcription of CaV1.1 in fast adult fibers. TnT-T3 becomes fractionated in aged muscle fibers, resulting in a reduced expression of CaV1.1 and the uncoupling of the ECC (Zhang et al., 2016). Preventing the TnT-T3 fragmentation maintained the CaV1.1 expression and EEC in old mice.

The CaVβ1 is a cytosolic subunit of DHRP that regulates the plasma membrane localization of the pore subunit CaVα1.1 as well as modulates the DHRP Ca^2+^ currents and is essential to form tetrads in skeletal muscles (Gregg et al., 1996; Hogan et al., 1999; Dayal et al., 2022). In addition to its role in channel regulation, CaVβ1 translocates to the nucleus, functioning as a transcriptional regulator during skeletal muscle development. The loss of CaVβ1 expression impaired the proliferation of muscle progenitor cells, resulting in underdeveloped muscles (Taylor et al., 2014). CaVβ1 expression increases with aging, causing a reduced expression of CaV1.1. The knock-down of CaVβ1 expression in old mice restores the charge movement in flexor digitorum brevis fibers to level of younger mice (Taylor et al., 2009). CaVβ1 is encoded by 13 exons of the 25 kb long *CACNA1B* gene. *CACNA1B* gene is subject to alternative splicing at exon 3 with an alternative start codon, exon 7, and exon 13 generating six splice variants CaVβ1-A, CaVβ1-B, CaVβ1-C, CaVβ1-D, CaVβ1-E (Traoré et al., 2019). CaVβ1-D is the adult skeletal muscle-specific alternative spliced isoform. The alternative splice variant CaVβ1-E is the embryonic variant. In the healthy adult muscle, CaVβ1-E is not expressed, but denervation switches the alternative splicing regulation, and its expression is upregulated. CaVβ1-E expression then plays a role in maintaining muscle mass. In old human and mouse muscles, the expression of CaVβ1-E is lost, contributing to aging-dependent muscle wasting. However, its overexpression in older mice counters the age-related muscle decline (Traoré et al., 2019).

Aging, thus, results in the repression CaV1.1 expression through the prevention *CACNA1S* gene transcription, resulting in a lower expression of L-type VGCCs in the T-tubule (Delbono et al., 1995; Zhang et al., 2016). This lower expression results in the uncoupling of the ECC, altering the force generation. Aging represses the alternative splicing mechanism of the CaVβ1 subunit, favoring the expression of the embryonic isoform expression, CaVβ1-E, in younger mice, which can contribute to muscle mass maintenance through its transcriptional regulator function (Traoré et al., 2019). The modulation of CaVβ1-E on the DHRP activity and T-tubule remains to be explored. The reduced expression of DHRP subunits in aging muscles impairs the ECC and contributes to muscle loss and weakness.

### Ryanodine receptor in aging skeletal muscle

The release of Ca^2+^ ions from the SR activates muscle contraction (Pearce et al., 2023). The Ryanodine Receptor type 1 (RYR1) is the Ca^2+^ channel on the SR membrane responsible for this Ca^2+^ release. The RYR1 cations permeable pore opens upon the sarcolemma depolarization that activates voltage-sensors movement of DHPRs physically connected to RYR1 channels (Figure 1).

### Ryanodine receptor’s structure and function

Ryanodine receptor (RYR) is a family of intracellular Ca^2+^ permeable channels formed by RYR1, RYR2, and RYR3 members. Skeletal muscles selectively express RYR1, cardiac muscle expresses RYR2, and the brain and other tissues, including the skeletal muscles, express RYR3 (Bauerová-Hlinková et al., 2020). RYR1 major function is the contraction of skeletal muscles (Pearce et al., 2023). RYR3 is highly expressed in neonatal stages in skeletal muscles. However, in adults, its expression is muscle-dependent (Protasi et al., 2000). RYR3 does not contribute to the excitation-contraction mechanisms. It has been shown that RYR3 does not restore the excitation-contraction coupling when expressed in RYR1-deficient myotubes (Fessenden et al., 2000; Ward et al., 2001). RYR3 mediates Ca^2+^ sparks in skeletal muscles (Ward et al., 2001; Pouvreau et al., 2007; Weisleder et al., 2007).

RYRs are the largest known ion channels. They are formed by a homotetramer of four identical subunits of about 5000 amino acids. Each monomer is composed of a transmembrane domain forming the channel cation pore of RYR1 and a large cytosolic domain with various binding sites for activators, such as Ca^2+^ ion, caffein or ATP, inhibitors, such as FKBP12 and FKBP12.6, (Chirasani et al., 2021; Yuchi et al., 2015), and modulator such as Mg^2+^ ions (Smith et al., 1986). The structure of RYR1 has been solved in closed, primed, open conformation and in complex with various modulators (Zalk et al., 2015; Yan et al., 2015; Des Georges et al., 2016). The structure of RYR3 was also recently solved (Chen et al., 2024). Located at the C-terminal end, the six transmembrane alpha-helices of each RYR monomer form the cation-permeable pore. The six alpha-helices (S1 to S6) are arranged similarly to the voltage-gated sodium channel. The S5 and S6 α-helices and the S5-S6 connecting loop (P-loop) form the pore and its selectivity filter, respectively. S5 alpha-helix was recently shown to contribute to the channel’s gating in addition to its pore-forming function (Murayama et al., 2024). The transmembrane α-helices S1 to S4 form a voltage-sensor-like structure (Zalk et al., 2015; Chen et al., 2024; Efremov et al., 2015). The N-terminal domain forms the large cytoplasmic moiety of RYR, containing various binding sites for modulators. The different subdomains of each homomer are A and B N-terminal, the N-terminal α-solenoid 1, bridging α-solenoid, the core α-solenoid, three SPRY (SPYR1, SPYR2, SPYR3), and the EF-hand. The C-terminal domain extends after the S6 α-helix binds to the core α-solenoid of the N-terminal domain of RYR. Ca^2+^ ions, Ryanodine, and ATP molecules bind at this interface region of the C-terminal domain, regulating RYR activation (Des Georges et al., 2016).

Several interacting proteins anchor RYR1 to specific locations or to partner proteins and regulate the activity. The major partner protein of RYR1 is the pore subunit of the DHPR, CaV1.1. The RYR1 and CaV1.1 couple constitute the Ca^2+^ release unit (CRU), the core of the excitation-contraction coupling. A CRU consists of four CaV1.1 proteins in contact with a single RYR1 channel, forming a tetrad (Xu et al., 2024). RYR1 and CaV1.1 interact at triad (also called diad) junctions, the close juxtaposition of T-tubule and SR membranes (Xu et al., 2024). Triad junctions are built with the help of Junctophilins (JPs) that form a connecting bridge between the T-tubule membrane and the SR membrane (Takeshima et al., 2000). JPs are also essential for the structural formation of the CRU by recruiting CaV1.1 at the triad (Nakada et al., 2018). The structure of a tetrad was recently observed by cryo-electron tomography (Xu et al., 2024). The resolution of the tetrad structure was solved at 33Å. RYR1 and CaV1.1 interact directly and through two binding partners, β1a and STAC3 (Dayal et al., 2022). β1a and STAC3 are essential for CaV1.1 trafficking and correct targeting at the plasma membrane to form the tetrade (Polster et al., 2015; Leuranguer et al., 2006). Precise control of RYR1 and CaV1.1 trafficking is essential for the formation of the CRU. Interestingly, a balanced expression of short- and long-alternative splice variant isoforms of four membrane trafficking regulators, synaptosomal-associated protein 23, the Cdc42-interacting protein 4, the clathrin heavy chain, and the transmembrane emp24 domain-containing protein 2, is crucial for the formation of the CRU (Giudice et al., 2016).

### Ryanodine receptor in aging

The loss of muscle force generation related to aging is linked to dysfunction of RYR1. The amount of the SR Ca^2+^ release is reduced with aging, lowering the force generation from aged muscles in mice (Jiménez-Moreno et al., 2008). Similarly, in humans, the Ca^2+^ release from SR and the force production are reduced in muscle fibers isolated from old humans compared to young humans (Lamboley et al., 2015; Lamboley et al., 2016). The reduced SR Ca^2+^ release is caused by a leaky RYR1 in old muscles, leading to a decreased amount of SR Ca^2+^ available. The passive RYR1 leak of Ca^2+^ has a physiological function in heat generation in resting muscles (Meizoso-Huesca et al., 2022). However, the passive RYR1 leak is also a cause of muscle dystrophy (Bellinger et al., 2009) and limitation in physical exercise capacity (Bellinger et al., 2008). In aging, RYR1 also leaks Ca^2+^ from SR and causes muscle weakness due to reduced available Ca^2+^ ions amount in the SR (Andersson et al., 2011). Interestingly, a common mechanism leads to the RYR1 Ca^2+^ leak in aging, muscle dystrophy, and reduced exercise capacities. Oxidative stress caused cysteine-nitrosylation of RYR1, turning it into a Ca^2+^ leaky channel. Oxidative stress also disrupts the binding of calstabin-1 (FKBP-12), a protein stabilizing RYR1 into its close conformation (Figure 1). The leak Ca^2+^ activates the Ca^2+^-activated protease calpain in the cytosol (Belcastro, 1993). Proteolysis of JP1 and JP2 by calpain might contribute to the disruption of the CRU (Murphy et al., 2013). Interestingly, preventing the uncoupling of calstabin-1 from RYR1 using the S107 molecule reduces the Ca^2+^ leak reactive oxygen species release and enhances the muscle force generation and exercise capacity (Andersson et al., 2011).

### Mechanically-gated channel Piezo1 in skeletal muscle

The activity of Ca^2+^-permeable mechanically-gated ion channels in skeletal muscle was first measured in 1990 (Franco and Lansman, 1990). The protein associated with this mechanical-dependent Ca^2+^ influx was recently identified as Piezo1, a mechanically gated ion channel. Although not highly selective to Ca^2+^ ions, the influx of Ca^2+^ mediated by Piezo1 has been recently shown to be essential in the myogenesis and maintenance of muscle satellite cells (Tsuchiya et al., 2018). Piezo channels form a two-member family of mechanically gated channels, Piezo1 and Piezo2. Piezo1 and Piezo2 are essential in transducing mechanical forces in somatosensation, proprioception, skin wound healing, and bones. An exhaustive review of Piezo channels’ physiological role was recently published by Xiao B (Xiao, 2024). Piezo1 is a large trimeric plasma membrane protein forming a three-flexible propeller (or blade) structure. Each monomer of Piezo1 is composed of 38 transmembrane domains, of which 36 transmembrane domains extend outward from the channel’s pore. Flexible propellers can curve the plasma membrane and regulate the channel’s gating. The pore on Piezo1 is formed by the last two C-terminal domain transmembrane α-helix (Saotome et al., 2018; Yang et al., 2022). Interestingly, Piezo1 plays an essential role in force generation through the tendon elastic energy but not skeletal muscle contraction. A gain of function mutation on the Piezo1 channel increases the elastic energy stored in tendons by 3 times in mice carrying the mutation compared to wild-type mice. Mice carrying this Piezo1 mutation have an enhanced physical performance compared to wild-type mice (Nakamichi et al., 2022). Piezo 1-dependent Ca^2+^ influx by yoda-1, a specific agonist, increases Ca^2+^ concentration in skeletal muscle satellite cells and myotubes but not in myofibers. Furthermore, stimulation of Piezo1 using yoda-1 does not enhance muscle force generation (Bosutti et al., 2021). Piezo1 is expressed in C2C12 myoblast cells and is essential for the fusion of myoblast and the formation of elongated myotube. In myoblasts, Piezo1 is activated by a phospholipid flippase complex composed of ATP11A (P-type adenosine triphosphatase) and its auxiliary subunit CDC50. The Ca^2+^ influx through Piezo1 activates the assembly of actomyosin filaments through the activation of the RhoA/ROCK signaling pathway to control a polarized elongation of myotube (Tsuchiya et al., 2018). Piezo1 was also shown to activate quiescent muscle satellite cells toward a responsive state necessary for skeletal muscle regeneration (Ma et al., 2022). Immobilization and physical inactivity lead to muscle atrophy, which is often the case in the elderly. Mice experimentally immobilized led to a loss of Piezo1 expression concomitant with lower basal Ca^2+^ levels in skeletal muscles. The immobilized mice showed an increase of Krueppel-like factor 15 (KLF15) expression that, in turn, stimulated interleukin-6’s (IL-6) expression and secretion, known to mediate muscle atrophy (Hirata et al., 2022). A loss-of-function mutation of Piezo1 is the cause of Prune-belly syndrome (Amado et al., 2024). Patients show congenital myopathy, particularly in the ventral abdominal wall. The mutated Piezo1 channel showed a significantly reduced open probability. Taken together, these studies show that Piezo1 activity is necessary to prevent muscle atrophy and regeneration which is essential during aging. However, the effect of aging and sarcopenia on Piezo1 expression and function remains yet to be explored.

### Store-operated Ca^2+^ entry channels: TRPC and Orai1 in aging skeletal muscle

**FIGURE 2:**
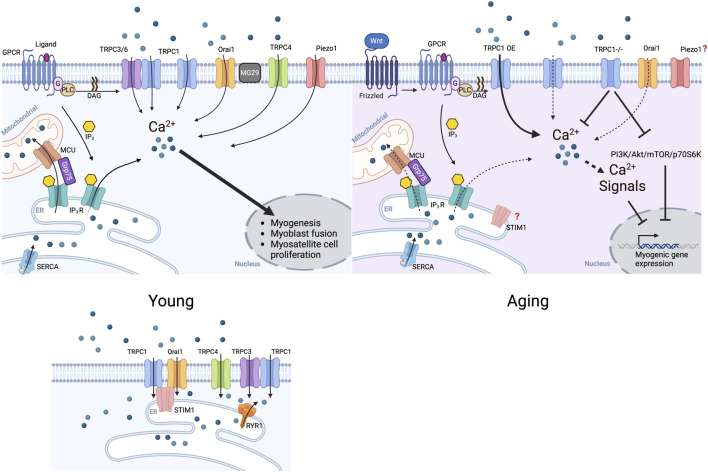
Ca^2+^ channels in skeletal muscle formation and regeneration. Schematic representation of Ca^2+^ channels expression and activity for muscle regeneration. TRPC channels and Orai1 mediate the store-operated Ca^2+^ entry at the plasma membrane to stimulate myogenesis, fusion, and myostatellite cell proliferation. Ca^2+^ influx mediated by Piezo1, the mechanically gated channel, contributes to myogenesis, particularly the fusion of myoblasts. TRPCs channels gating are regulated by STIM1 and RYR1. STIM1 stimulates Orai1 gating. TRPC channels’ expression lowers during aging reducing the store-operated Ca^2+^ entry and affecting myogenesis. The effect of aging on Orai1 and Piezo1 remains unexplored. The intracellular channels IP3R release calcium from the ER also contribute to myogenesis.

Store-Operated Ca^2+^ Entry (SOCE) is a fundamental pathway for Ca^2+^ influx in virtually all mammalian cell types, playing a critical role in intracellular signaling and Ca^2+^ homeostasis (Hogan and Rao, 2015). SOCE is particularly important in cells requiring sustained Ca^2+^ influx for prolonged and/or repeated signals, including neurons, muscle, and immune cells (Avila-Medina et al., 2018; Bollimuntha et al., 2017; Feske, 2012). SOCE functions in the immune system in, for example, T-lymphocytes and mast cells, where Ca^2+^ signaling is essential for their activation, proliferation, and cytokine production (Vaeth et al., 2017; Ma et al., 2011). In skeletal muscle, SOCE is pivotal in maintaining Ca^2+^ homeostasis, facilitating muscle formation and regeneration, and supporting sustained muscle contraction (Figure 2). (Lilliu et al., 2021; Koenig et al., 2019).

In skeletal and cardiac muscle, SOCE helps maintain Ca^2+^ levels during repeated or sustained contractions by replenishing Ca^2+^ stores in the SR (Lilliu et al., 2021). As Ca^2+^ is released from the SR during the ECC, SR Ca^2+^ stores get depleted. This depletion of Ca^2+^ ions concentration in SR activates the SOCE at the plasma membrane. STIM (Stromal Interaction Molecule) is an SR membrane protein with a Ca^2+^ sensing domain in the ER/SR lumen. In the absence of Ca^2+^, STIM undergoes a self-oligomerization and expands to reach the plasma membrane interacting with Orai1 and/or TRPC channels. The interaction of STIM1 with Orai1 and/or TRPC gates the channels, allowing the Ca^2+^ entry (Avila-Medina et al., 2018). This flow of Ca^2+^ ions from the extracellular space increases the intracellular Ca^2+^ concentration and enables replenishment of SR Ca^2+^ stores with the activity of the Sarcoplasmic-Endoplasmic Reticulum Calcium ATPase (SERCA) pumps. Thus, SOCE ensures that Ca^2+^ influx across the plasma membrane is maintained, particularly when internal stores are depleted, sustaining proper muscle function during repeated or prolonged contractions (Figure 2).

Dysfunction of the SOCE is linked to several muscle-related diseases, some associated with aging. One such condition is sarcopenia, the rapid progressive loss of muscle mass and strength occurring with aging (Brotto, 2011; Protasi et al., 2023). The increase in intracellular Ca^2+^ is crucial for activating myosatellite cells and regulating myoblast migration and fusion during myogenesis and muscle regeneration, which is essential for proper muscle repair after injury, muscle growth, and recovery after atrophy (Sinha et al., 2022; Tu et al., 2016). Impaired SOCE leads to disruptions in Ca^2+^ homeostasis, which affects muscle formation and regeneration, contributing to the weakening of skeletal muscles (Sztretye et al., 2017). SOCE dysfunction is also implicated in Age-Related Myositis and plays a role in Duchenne Muscular Dystrophy (DMD), a genetic disorder characterized by muscle degeneration. In these conditions, the impaired regulation of Ca^2+^ entry may lead to muscle damage, inflammation, and decreased muscle repair capacity (Mareedu et al., 2021). In DMD, the absence and dysfunction of dystrophin makes muscle fibers more susceptible to injury during contraction. Ca^2+^ overload followed by improper SOCE, exacerbates the damage upon proteases activation, such as calpains. These all degrade muscle proteins and cause inflammation (Millay et al., 2009; Gailly et al., 2007). Over time, the sustained inflammatory response further damages muscle tissue, leading to fibrosis and impaired muscle function. Furthermore, reduced SOCE function has been reported to depress muscle regeneration, a key impact factor in aging and muscular dystrophies (Thornton et al., 2011). In muscular dystrophies, this results in the replacement of healthy muscle tissue with fibrotic or fatty tissue, further reducing muscle mass, strength, and function (Piñol-Jurado et al., 2024).

### TRPC channels’ structure and function in skeletal muscle

TRPC channels belong to TRP (Transient Receptor Potential) channel family. The TRP family is subdivided into six subfamilies based on sequence homology and functional characteristics: TRPC (Canonical), TRPV (Vanilloid), TRPM (Melastatin), TRPA (Ankyrin), TRPML (Mucolipin), TRPP (Polycystin) (Blair et al., 2023). Each subfamily has distinct physiological roles, but they all share the core structure of six transmembrane domains (S1-S6) with a pore-forming region between the fifth and sixth segments. The pore loop determines the channel’s ion permeability and selectivity. Although TRP channels are non-selective for cations, their Ca^2+^ permeability enables the generation of essential intracellular Ca^2+^ signals (Blair et al., 2023; Owsianik et al., 2006). TRPC channels consist of seven members, TRPC1 to TRPC7. Several TRPC members play a role in muscle physiology and pathology, including the cardiovascular system and smooth muscles (Choi et al., 2020; Gonzalez-Cobos and Trebak, 2010; Freichel et al., 2017). In skeletal muscles, some TRPC members can regulate Ca^2+^ dynamics in response to various stimuli, mechanical stretch, and SR Ca^2+^ depletion (Choi et al., 2020; Formigli et al., 2009; Zanou et al., 2010).

TRPC1, TRPC3, TRPC4, and TRPC6 are consistently expressed in cultured myoblasts or adult skeletal muscle (Gailly, 2012; Antigny et al., 2013). TRPC1 is the predominant isoform expressed in skeletal muscle, smooth muscle, and endothelial cells which can regulate Ca^2+^ influx as a SOCE channel to support processes like prolonged muscle contraction and vascular tone (Ahmmed and Malik, 2005; Wen et al., 2020). When myogenesis was stimulated in the C2C12 myoblast cell line, the expression of TRPC1 transiently increased after 24 h of differentiation and then returned to a basal expression value from day 4 to day 6. The developmentally regulated increase at the beginning of differentiation is necessary for myoblast migration and fusion stages (Louis et al., 2008). TRPC1 involves both mechano-sensation and SOCE, which is crucial for refilling intracellular Ca^2+^ stores and responding to mechanical stimuli such as stretch, particularly during skeletal muscle contraction, myoblast migration, and differentiation (Formigli et al., 2009; Louis et al., 2008; Antigny et al., 2017). In myoblasts, TRPC1 is an essential stretch-activated channel modulated by sphingosine 1-phosphate, a bioactive lipid in satellite cells and myogenesis (Formigli et al., 2009). When TRPC1 was knocked down in myoblasts, SOCE and the calpain (a Ca^2+^-dependent protease, which shows peak activity at the onset of differentiation) activity were significantly reduced, leading to the accumulation of MARCKS (an actin-binding protein) and slower cell migration and fusion (Louis et al., 2008).

TRPC3 is expressed in mouse skeletal myoblasts and shows an initial peak during the early stage of differentiation, followed by a gradual decline as differentiation progresses (Woo et al., 2008). Ca^2+^ entry through TRPC3 via the SOCE mechanism in C2C12 myotubes activates the CaN (calcineurin)/NFAT signaling pathway, which is essential in determining myotube phenotype. This activation shifts gene expression from a fast-glycolytic to a slow-oxidative phenotype in a CaN-dependent manner, promoting muscle fiber transitions from endurance and resistance to fatigue (Rosenberg et al., 2004). Additionally, TRPC3 interacts functionally with RYR1, which is crucial for SR Ca^2+^ release during ECC. However, TRPC3 does not directly bind to RYR1, and the interaction requires a protein linker. Six triadic proteins as a physical link between TRPC3 and RYR1 can regulate RYR1 function, and ECC: TRPC1, junctophilin 2, homer, mitsugumin 29, calreticulin, and calmodulin. Among these, TRPC1 was identified as a potential physical link between TRPC3 and RYR1 (Woo et al., 2008; Woo et al., 2014).

TRPC4 is involved in SOCE with TRPC1, which is necessary to activate the transcription factor myocyte enhancer factor-2 (MEF2). MEF2, in synergy with MyoD, facilitates the fusion of human myoblasts into myotubes (Antigny et al., 2013). During mouse and human myoblast differentiation, silencing or dominant-negative suppression of TRPC1 and TRPC4 reduces SOCE, leading to thin and short myotubes with fewer nuclei (Formigli et al., 2009; Antigny et al., 2017; Sabourin et al., 2009). In myotubes, TRPC1 and TRPC4 work together with STIM1L, abundantly expressed in skeletal muscles, to maintain fast repetitive Ca^2+^ influx and proper differentiation program (Antigny et al., 2017).

The role of TRPC6 in skeletal muscle remains unexplored, despite its known expression in this tissue (Zanou et al., 2010). There is no research on TRPC2 function in skeletal muscles, as it is only expressed in non-human mammals and is essential in pheromone detection (Zufall, 2005). Additionally, the expression levels of TRPC5 and TRPC7 are lower than other TRPC subtypes in skeletal muscle, highlighting the need for further studies to clarify their roles (Zanou et al., 2010).

TRPC isoforms form tetrameric structures with either four identical subunits (homotetramers) or a combination of different TRPC subunits (heterotetramers). These homo- and heterotetrameric isoforms generate functional ion channels. TRPC3 can form heteromers with TRPC1 via its N-terminal ankyrin repeats and regulates the cytosolic Ca^2+^ levels for the ECC of skeletal muscle (Woo et al., 2014; Cheung et al., 2011).

### Role of TRPC channels in aging skeletal muscle

With aging, the function and expression of TRPC1 are altered, which affects Ca^2+^ homeostasis and leads to muscle dysfunction. Reduced TRPC1 activity in aging muscles is associated with impaired Ca^2+^ handling, resulting in a decreased prolonged contraction capacity and increased muscle fatigue (Zanou et al., 2010). The role of TRPC1-mediated Ca^2+^ entry is strongly linked to muscle fatigue. Muscles from TRPC1 knockout mice show progressively lower Ca^2+^ transients during repeated stimulation compared to WT fibers, accompanied by a significant force loss. TRPC1 knockout mice also display smaller muscle fiber cross-sectional areas, generate less force per area, and contain fewer myofibrillar proteins, though no other signs of myopathy are present. These mice also experience a notable decrease in physical endurance. In DMD, abnormal TRPC1 activation may involve ROS production and src kinase activation, further impacting muscle function (Gervásio et al., 2008). The PI3K/Akt/mTOR/p70S6K pathway, essential for muscle growth and regeneration, is downregulated in TRPC1-deficient muscles, with reduced phosphorylation of Akt and p70S6K, and decreased PI3K activity. In TRPC1-deficient myoblasts or myoblasts lacking extracellular Ca^2+^, Akt phosphorylation is also reduced (Zanou et al., 2012).

The phenotype resulting from TRPC3 overexpression closely resembles muscular dystrophy, not myopathy. Overexpression of TRPC3 and the resulting increase in Ca^2+^ influx led to dystrophic conditions like fibrosis, fatty tissue replacement, myofiber degeneration, cycles of regeneration, and immune cell infiltration. Inhibition of TRPC channels in mice reduced Ca^2+^ influx and alleviated dystrophic symptoms associated with mdx mutation (*dystrophin* gene) and *Scgd* gene deletion (*α-sarcoglycan* gene) (Millay et al., 2009).

The dysregulation of TRPC channels in aging could impair muscle performance, but their modulation presents a potential therapeutic avenue. TRPC1 overexpression enhances β-catenin expression and promotes myogenesis. TRPC1 facilitates the accumulation of intracellular Ca^2+^ and the nuclear translocation of the NFATC2/NFATC2IP complex. It further promotes muscle growth by the Wnt/Ca^2+^ pathway, suggesting that targeting TRPCs could be a viable strategy for combating age-related muscle decline (Hao et al., 2024).

### Orai1’s structure and function in skeletal muscle

Orai1 is a member of the Orai family of Ca^2+^ channels, which includes Orai2 and Orai3. These channels are key components of SOCE in various tissues, but Orai1 is especially prominent in excitable tissues like muscle and immune cells (Rubaiy, 2023). Orai1 is a transmembrane protein that forms a hexameric channel in the plasma membrane, with each of the six subunits contributing to the central pore. The pore contains a Ca^2+^ selectivity filter formed by conserved glutamate residues that ensure selective Ca^2+^ influx (Hou et al., 2012).

Orai1 is directly responsible for SOCE, once activated by the drop in SR Ca^2+^ level, STIM1 proteins cluster together and move toward specific regions of the SR membrane close to the plasma membrane. STIM1 cytosolic domain then reaches out to interact with Orai1 by its SOAR (STIM1 Orai-activating region) and triggers a structural change in Orai1, causing the channel to open (Yuan et al., 2009). Orai1 acts as the “door” that opens to allow Ca^2+^ to flow into the cell after Ca^2+^ stores depletion, working with STIM1 to maintain the intracellular Ca^2+^ homeostasis.

In adult muscle fibers, SOCE is abolished in mice with either muscle-specific expression of a dominant-negative Orai1 or a muscle-specific Orai1 knockout (Carrell et al., 2016; Wei-LaPierre et al., 2013). In skeletal muscle fibers, the T-tubules membrane containing Orai1 elongates into the I-band, towards the Z-line, forming junctions with SR membranes containing STIM1 proteins. Exercise promotes new SR-Tubules junctions in the I-band, facilitating STIM1/Orai1 interactions to boost Ca^2+^ entry and muscle performance during intense activity. STIM1 remains primarily within the I-band after exercise, while some Orai1 translocate toward the Z-line, increasing STIM1/Orai1 co-localization (Orci et al., 2009; Perni et al., 2015). This remodeling enhances SOCE, in turn, muscles can become more resistant to fatigue during high-frequency stimulation (Boncompagni et al., 2017). In the constitutive muscle-specific Orai1 knockout mice, normal postnatal growth and fiber-type differentiation were observed initially. Then as age, a significant reduction in muscle fiber cross-sectional area was evident, particularly in oxidative, fatigue-resistant fiber types. These mice showed a decrease in type I muscles fibers, along with an increase in hybrid fibers expressing both type I and type IIA myosin. Additionally, the mice exhibited reduced maximal specific force and endurance (Carrell et al., 2016).

### Role of Orai1 in aging skeletal muscle

Abnormal upregulation of the STIM1 – Orai1 complex is implicated in a wide range of muscle diseases, from muscular dystrophies to sarcopenia. Orai1-STIM1 dysregulation leads to Ca^2+^ overload in dystrophic mouse muscle cells (Goonasekera et al., 2014). In DMD, abnormal activation of Orai1 and STIM1 causes excessive Ca^2+^ influx as well (Uchimura and Sakurai, 2021). This constant Ca^2+^ entry triggers cell death (apoptosis) and muscle degeneration (Goonasekera et al., 2014; Burr and Molkentin, 2015). Muscle fibers might lose their ability to regenerate effectively, worsening muscle weakness and damage (Uchimura and Sakurai, 2021). Orai1 knockout in the DMD mouse model reduced Ca^2+^ overload, prolonged the electrically evoked Ca^2+^ transient decay rate, improved muscle regeneration, and delayed disease progression (García-Castañeda et al., 2022). A loss-of-function mutation in Orai1 was identified in patients with combined immunodeficiency (CID) along with severe skeletal muscle myopathy (tubular aggregate myopathy (TAM), muscular hypotonia) (Lacruz and Feske, 2015). A report on this mutation showed a predominance of type I muscle fibers and atrophy of type II fibers, suggesting that Orai1 supports the maintenance of muscle fiber types (McCarl et al., 2009).

A reduction in STIM1/Orai1-mediated SOCE is believed to contribute to the decline in muscle contractile force and increased fatigue in aging. Recent studies have linked SOCE to muscle weakness during aging. SOCE is significantly compromised in muscle fibers from aged (26–27 months) mice compared to young (2–5 months) mice. However, this reduction in SOCE is not due to altered STIM1 or Orai1 expression but is linked to decreased expression of mitsugumin-29, a synaptophysin-related protein that plays a role in SOCE regulation. Reduced mitsugumin-29 and compromised SOCE may contribute to impaired Ca^2+^ homeostasis in aging muscle, leading to weakness (Thornton et al., 2011; Zhao et al., 2008). Thus, the specific role of STIM1/Orai1-dependent SOCE in the age-related decline of skeletal muscle performance remains unclear. Further research is necessary to fully understand this relationship.

### Inositol 1,4,5-trisphosphate receptor in aging skeletal muscle

#### IP3R’s structure and function in skeletal muscle

The IP3R (Inositol 1,4,5-trisphosphate receptor) is a large intracellular Ca^2+^ release channel primarily located on the membrane of the endoplasmic reticulum (ER) or sarcoplasmic reticulum (SR). Three different genes (*ITPR1*, *ITPR2*, and *ITPR3*) exist in the human genome, giving rise to the corresponding proteins (IP3R1, IP3R2, and IP3R3) (Taylor et al., 1999). It has a complex quaternary structure, and each receptor is typically formed by four subunits, creating a tetrameric architecture. The functional IP3R channel comprises four identical or similar subunits arranged symmetrically around a central pore. The transmembrane domain consists of six transmembrane α-helices per subunit, with the fifth and sixth α-helices forming the Ca^2+^-selecting pore. Large cytoplasmic regions on the N- and C-termini contain sites for binding various regulatory proteins and molecules, including Ca^2+^ itself, ATP, and other modulators (Prole and Taylor, 2019). These domains are essential for fine-tuned control over Ca^2+^ release, depending on cellular conditions (Schmitz et al., 2022). When a ligand binds to a G-protein-coupled receptor (GPCR), a Gq-protein can be activated, then, in turn, activate the phospholipase C (PLC) enzyme. PLC cleaves a membrane phospholipid, PIP2, into inositol 1,4,5-trisphosphate (IP3) and diacylglycerol (DAG). IP3 molecules diffuse through the cytoplasm and bind to IP3R on the ER and SR, triggering the release of Ca^2+^ into the cytosol. This Ca^2+^ concentration increase regulates muscle contraction, cytoskeleton remodeling, and secretion. Meanwhile, DAG activates protein kinase C (PKC), initiating additional signaling pathways (Wu and Chen, 2023). The expression pattern and subcellular distribution of the three IP3R isoforms show heterogeneity in different tissues and cell types (Prole and Taylor, 2016; Monkawa et al., 1995).

IP3R1-mediated Ca^2+^ release is critical for neuromuscular junction (NMJ) development, synaptic gene expression, and neuromuscular transmission (Powell et al., 2003). Inhibition of IP3R1 in C2C12 cells reduced calpain activity and prevented acetylcholine receptor (AChR) cluster dispersal. In adult NMJs, IP3R1 knockdown increased synaptic strength and AChR expression. In mouse models of cholinergic hyperactivity, such as slow channel myasthenic syndrome (SCS), IP3R1 knockdown prevented NMJ Ca^2+^ overload, protease activation, and DNA damage, improving neuromuscular function (Zhu et al., 2011). Dystrophin-deficient myotubes exhibit enhanced IP3-mediated Ca^2+^ signaling at rest, with increased release site density. The heightened Ca^2+^ release may contribute to Ca^2+^ overload in muscle cells (Balghi et al., 2006). IP3R activity is crucial for slow Ca^2+^ waves and ERK1/2 phosphorylation in mouse skeletal muscle. The Ras-ERK signaling pathway is a key factor for muscle stimulation and gene expression, particularly in myosin gene expression during muscle regeneration (Murgia et al., 2000). The brief depolarization can rapidly trigger ERK phosphorylation, independent of extracellular Ca^2+^, and this response is blocked by the IP3R inhibitor 2-APB. This suggests a direct connection between IP3R-mediated Ca^2+^ signals and MAP kinase activation in skeletal muscle (Powell et al., 2001).

#### Role of IP3R in aging skeletal muscle

In aging mice’s skeletal muscle, IP3R1 expression significantly decreases, impairing myotube formation and muscle regeneration. This decline results from the repression of muscle-specific genes and activation of the EGFR-Ras-ERK pathway (Choi et al., 2016). The conserved reduction in IP3R1 in human aging suggests it is a potential therapeutic target for sarcopenia, with ERK inhibition as a promising treatment strategy.

IP3R and RYR work together to regulate Ca^2+^ dynamics during the ECC. RYR is primarily responsible for Ca^2+^-induced Ca^2+^ release (CICR). The DHPR clusters at the triads are found on the margins of the IP3R-staining I-band of the SR in the mature myotube in culture (Powell et al., 1996). IP3R, activated by IP3 binding, modulates Ca^2+^ release from the SR, complementing the RYR’s function. In cardiomyocytes, IP3R activity in dyads increases the RYR-mediated Ca^2+^ spark formation (Chung et al., 2023). In pulmonary arterial smooth muscle cells (PASMC), IP3R interacts with other ion channels, such as the Ca^2+^-activated chloride channel (ANO1) and VGCC CaV1.2, to facilitate Ca^2+^ release from the SR, crucial for generating oscillating Ca^2+^ waves that drive vascular contraction (Akin et al., 2023). Both RYR1 and IP3R are essential for the mitochondrial Ca^2+^ increase triggered by membrane depolarization in skeletal muscle fibers, whether induced by potassium or electrical stimulation. This Ca^2+^ influx links muscle excitation to enhanced mitochondrial metabolic output, known as excitation-metabolism coupling (Díaz-Vegas et al., 2018). IP3R overactivity in mdx contributes to elevated mitochondrial Ca^2+^ levels, disrupts mitochondrial dynamics, and increases autophagy and mitophagy, all of which normalize when IP3R is downregulated (Valladares et al., 2018). SR-mitochondria Ca^2+^ transport proteins IP3R1, Grp75, and VDAC are downregulated and the IP3R encoding gene expression is reduced in the skeletal muscle of aged mice (Gill et al., 2019). Overactive IP3R leads to excessive Ca^2+^ release from the SR, causing chronic Ca^2+^ overload in the cytosol and mitochondria. This disrupts autophagy and mitochondrial function, contributing to muscle degeneration (Figure 2) (Decuypere et al., 2011). Since proper IP3R function is essential for mitochondrial bioenergetics and ATP production, the decline in IP3R activity during ER stress or reduced IP3 signaling may play a significant role in age-related apoptosis, though further research is required.

### Mitochondrial Ca^2+^ channels in aging skeletal muscle

#### Mitochondrial in different skeletal muscle fibers

In muscle tissues, mitochondria exhibit significant differences between fiber types. Fast-twitch fibers (Type II), which rely on anaerobic respiration, contain fewer mitochondria and blood vessels, allowing rapid contraction but leading to quick fatigue. Conversely, slow-twitch fibers (Type I), which depend on aerobic respiration, have a higher mitochondrial content and a denser network of blood vessels, supporting sustained energy production and endurance (Henneman and Olson, 1965; Jackman and Willis, 1996). Endurance training causes mitochondrial adaptations in muscles, including the expansion of the mitochondrial network, especially in slow-twitch fibers. These fibers show higher rates of mitochondrial fusion and increased cristae density, improving oxidative capacity and enabling muscles to enhance energy production efficiency (Kirkwood et al., 1987; Nielsen et al., 2017). Key regulatory pathways like AMPK-SIRT1-PGC-1α and IGF-1-PI3K-Akt-mTOR influence the progression of mitochondrial dysfunction, impacting muscle mass and quality (Kubat et al., 2023).

#### Role of mitochondrial Ca^2+^ channels in dysfunctional and aging skeletal muscle

Mitochondrial dysfunction is widely recognized as an indicator of skeletal muscle atrophy. One key contributor to this dysfunction is the Ca^2+^ overload that occurs through ER–mitochondrial contacts. These contacts, also known as mitochondria-associated membranes (MAMs), facilitate the transfer of Ca^2+^ from the ER into mitochondria, playing a critical role in cellular homeostasis but becoming problematic during aging and in muscle diseases (Lv et al., 2022). Mutations in RYR1, linked to muscle disorders like malignant hyperthermia (MH) and central core disease (CCD), increase the channel’s opening probability, resulting in excessive Ca^2+^ release into the cytoplasm (Wu et al., 2006; Iyer et al., 2022). The depletion of SR Ca^2+^ activates SOCE, which allows Ca^2+^ influx from the extracellular space. This mechanism becomes upregulated in DMD and MH, further increasing Ca^2+^ levels in the myoplasm (Bround et al., 2024; Duke et al., 2010). The Ca^2+^ is then taken up by mitochondria through the mitochondrial Ca^2+^ uniporter (MCU). Excessive mitochondrial Ca^2+^ uptake leads to a Ca^2+^ overload, which disrupts mitochondrial function by triggering an overproduction of reactive oxygen species (ROS) and reactive nitrogen species (RNS). This exacerbates oxidative stress, damaging mitochondrial components and impairing their role in energy production (Michelucci et al., 2021). Increased ROS/RNS production leads to oxidative damage to proteins, lipids, and DNA, contributing to the progressive decline in muscle function (Lian et al., 2022). The Ca^2+^ overload also represses ATP production, contributing to muscle fatigue and the gradual loss of muscle mass in sarcopenia and dystrophies. Aged muscle cells experience an increase in resting mitochondrial Ca^2+^ levels, leading to enhanced mitophagy for removing damaged mitochondria. MCU inhibition in *Caenorhabditis elegans* model of muscular dystrophy reduces excessive mitochondrial Ca^2+^, improving movement (Higashitani et al., 2023; Hewitt et al., 2018).

Moreover, skeletal muscle-specific MCU deletion reduces mitochondrial Ca^2+^ uptake and triggers a systemic catabolic response, leading to impaired muscle force and reduced exercise performance (Gherardi et al., 2019). As a gatekeeper, Mitochondrial calcium uptake family member 1 (MICU1) works to fine-tune MCU activation and maintain threshold control. It regulates Ca^2+^ influx through MCU, preventing uptake at low cytosolic levels and enabling it when Ca^2+^ is elevated. Loss of MICU1 in skeletal muscle disrupts mitochondrial Ca^2+^ uptake during ECC, impairing aerobic metabolism and resulting in reduced contractile force, muscle weakness, and increased fatigue (Debattisti et al., 2019). MICU3, a regulator of MCU from mitochondrial calcium uptake family expression, declines with aging in mouse skeletal muscle, leading to reduced mitochondrial Ca^2+^ uptake (Yang et al., 2021). Reduced mitochondrial Ca^2+^ levels result in increased pyruvate dehydrogenase phosphorylation and lower net NADH oxidation during muscle contractions, indicating disrupted energy metabolism. These are linked to impaired myogenesis, increased oxidative stress, and apoptosis. Additionally, the loss of MICU3 reduces time to fatigue and impairs exercise capacity by shifting muscle fiber composition from type IIa (high oxidative) to type IIb (low oxidative) fibers (Yang et al., 2021; Roman et al., 2024). These all suggest that mitochondrial Ca^2+^ imbalance plays a role in muscle degeneration during aging and dystrophy, highlighting MCU as a potential target for muscle loss intervention. Parvalbumin (PV), as a cytosolic Ca^2+^-binding protein, is highly expressed in fast-twitch skeletal muscle. PV upregulation leads to atrophy, while acute downregulation is associated with muscle hypertrophy. These are linked to increased mitochondrial Ca^2+^ uptake and enhanced interactions with Ca^2+^ release sites. Notably, silencing the MCU abolishes the hypertrophic effect of PV ablation (Butera et al., 2021). Therefore, PV may regulate muscle quality and mass by functionally interacting with MCU to modulate Ca^2+^ dynamics and mitochondrial adaptations. In addition, mice lacking Mcub (Mitochondrial calcium uniporter dominant-negative subunit beta), an inhibitor of MCU-mediated Ca^2+^ influx in skeletal muscle, exhibited increased pyruvate dehydrogenase activity, reduced fatty acid utilization, glucose intolerance, and increased adiposity (Huo et al., 2023). Pyruvate dehydrogenase kinase 4 (PDK4) regulates mitochondrial pyruvate dehydrogenase activity, and its increased expression is linked to aging muscle. Genetic removal of PDK4 can rescue muscle regeneration, prevent dexamethasone-induced muscle atrophy, and preserve muscle fibers (Perez et al., 2022; Sinam et al., 2022). Additionally, PDK4 interacts with and stabilizes the IP3R1-GRP75-VDAC1 complex at the MAM interface, influencing mitochondrial Ca^2+^ dynamics (Thoudam et al., 2019). These findings suggest that PDK4 could be a potential therapeutic target for treating muscle atrophy and age-related lipid metabolism dysfunction by influencing mitochondrial Ca^2+^ dynamics.

As individuals age, several significant changes occur in skeletal muscle, including a shift from type II to type I fibers. This transformation is often accompanied by increased fat deposition within and between muscle tissues, further impairing muscle quality. Additionally, the number of satellite cells, essential for muscle repair and regeneration, declines, particularly affecting type II fibers. Aging also results in alterations in mitochondrial structure and function within muscle cells, contributing to decreased energy production and increased oxidative stress (Cheng et al., 2023; Deschenes, 2004). In skeletal muscle, Ca^2+^ is critical for ECC, which involves the release of Ca^2+^ from the SR through channels like RYR1. This Ca^2+^ signaling becomes less efficient during aging, leading to improper muscle contraction, increased ROS production, and mitochondrial damage. Ca^2+^ also enters the mitochondria, regulating ATP synthesis and ROS levels (Hood et al., 2019). The accumulation of ROS in muscle cells can harm mitochondria, largely due to a decline in cellular antioxidant enzyme levels with aging. The cytosolic antioxidant enzyme CuZn-superoxide dismutase (CuZnSOD) is reduced in aged mouse nerves and muscle, causing sarcopenic muscle loss due to oxidative stress (Zhang et al., 2013). Aging cells exhibit increased susceptibility to the opening of the mPTP, a channel sensitive to Ca^2+^. Frequent opening of the mPTP can lead to loss of mitochondrial membrane potential, ATP depletion, and cell death, contributing to sarcopenia in aging muscles (Rottenberg and Hoek, 2021). The excessive mitochondrial Ca^2+^ uptake in isolated muscle fibers can be inhibited by cyclosporine A (CsA), an inhibitor of the mPTP regulator Cyclophilin D. CsA treatment improves Ca^2+^ handling, reduces ROS production during aortic cross-clamping, and restores mitochondrial function, as well as helps prevent muscle weakness (Pottecher et al., 2013; Gineste et al., 2015).

The decline in sex hormones, such as 17β-estradiol and testosterone, in aging, is linked to sarcopenia. These hormones play a role in maintaining mitochondrial function, and their reduction can impair mitochondrial regulation (Tian et al., 2023a). 17β-estradiol also appears to regulate mitochondrial function, including Ca^2+^ handling. Sex differences in vasodilation are partly due to variations in mitochondrial Ca^2+^ uptake. Female mice show enhanced Ca^2+^ buffering and mitochondrial function in endothelial cells, attributed to increased mitochondrial mass and membrane potential (Damacena et al., 2022). The differing rates of decline in these sex hormones with age may be a reason why muscle atrophy is more severe in men than in women among people over 60 years old.

Mitofusin 2 (Mfn2) is essential for protecting muscle mitochondria from damage, and its levels progressively decline with aging in mouse skeletal muscle as well. In Mfn2 knockdown in FDB (flexor digitorum brevis) muscle fibers, mitochondrial Ca^2+^ uptake was significantly reduced, correlating with an increase in the global myoplasmic Ca^2+^ transient. Additionally, Mfn2 knockdown decreased mitochondrial membrane potential, contributing to the observed impairment in activity-dependent mitochondrial Ca^2+^ uptake (Ainbinder et al., 2015). Additionally, overexpression of PGC-1α enhances Ca^2+^ buffering capacity and homeostasis in fast-twitch muscle, both at rest and after fatiguing tetanic contractions. This effect is associated with increased Ca^2+^ uptake potential, driven by the upregulation of Mfn1, Mfn2, and MCU, which improve intracellular Ca^2+^ homeostasis independently (Eshima et al., 2017).

### Preventing the effects of aging on skeletal muscles’ calcium channels

Maintenance of functional Ca^2+^ homeostasis, ECC, and Ca^2+^ signals for functional skeletal muscles is essential for the elderly to keep an independent life and lift the burden of care. Muscle atrophy and sarcopenia are major causes of increasing weakness while aging. Regular physical training, caloric restriction, natural bioactive compounds, and molecules are known to improve muscle mass and force generation and to prevent or reverse sarcopenia and age-related atrophy.

#### Effects of physical exercise training and caloric restriction on aging

**FIGURE 3:**
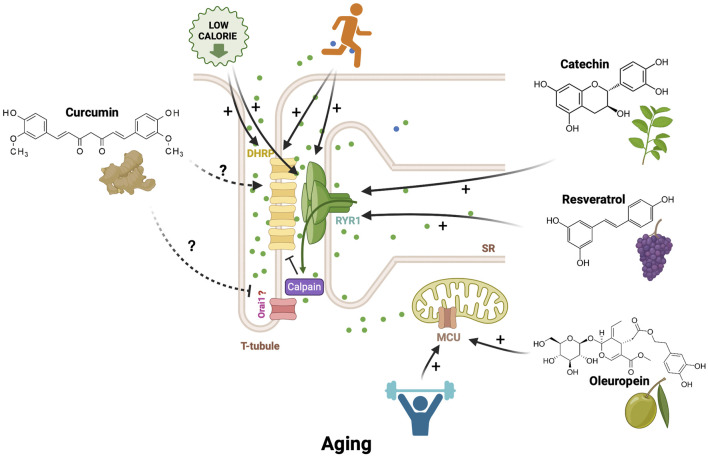
Bioactive compounds, caloric restriction, endurance, and resistance exercise modulate Ca^2+^ channels. Endurance exercise and caloric restriction enhance expressions of DHRP and RYR1. Resistance exercise and oleuropein (a phenolic compound from olives) increase the Ca^2+^ uptake in mitochondria, enhancing mitochondrial function in aged skeletal muscles. Catechins extracted from green tea leaves directly increase the activity of RYR1 and potentiate the Ca^2+^ transient in flexor digitorum brevis fibers. Resveratrol (a polyphenol molecule from grapevines) stimulates the inclusion of RYR1 exon 83, which is typically absent in Myotonic Dystrophy type I (DM1). Curcumin (a compound from turmeric) positively or negatively regulates various ion channels in rat hippocampal neurons, suggesting its potential clinical use in aging skeletal muscle.

Regular physical training attenuates aging muscle loss. Resistance training and electrical stimulation have been shown to prevent muscle atrophy and weakness in older adults (McKendry et al., 2024; Zampieri et al., 2015). Physical exercise training, both endurance and resistance, in elderly people stimulates the reinnervation of skeletal muscles and an increased expression of the RYR1 mRNA and the MCU mRNA and protein (Coletti et al., 2022; Bolotta et al., 2020; Zampieri et al., 2016). Endurance treadmill training in rats and mice increased DHRP and RYR1 protein expression in skeletal muscles (Saborido et al., 1995; Ferreira et al., 2010). In old rats (24 months), 6 weeks of regular endurance exercise training increased the expression of RYR1 (Figure 3). (Tromm et al., 2016).

Caloric restriction in old rats prevents the age-related reduction of DHPR and RYR1 expression and the uncoupling of RYR1 and DHRP (Renganathan and Delbono, 1998; Mayhew et al., 1998). How caloric restriction prevents this uncoupling remains to be investigated (Figure 3).

#### Effects of natural bioactive compounds on aging

The effects of natural bioactive compounds from plants and food on skeletal muscle are being extensively studied (Yadav et al., 2022). Some of these natural bioactive compounds have been shown to regulate Ca^2+^ channels’ activity and expression in multiple tissues, including skeletal muscles (Figure 3). Curcumin is a compound extracted from the turmeric (*Curcuma longa*) plant (Ayub et al., 2024). Preclinical studies reviewed in Gany SLS have shown that curcumin is a potential therapeutic molecule against sarcopenia (Saud Gany et al., 2023). Curcumin positively or negatively regulates various ion channels and transporters (Tabeshpour et al., 2019). In rat hippocampal neurons, curcumin inhibits the voltage-gated L-type Ca^2+^ currents through a PKC-dependent pathway (Liu et al., 2013). In bovine adrenal zona fasciculata, curcumin enhances the T-type voltage-gated Ca^2+^ currents (Enyeart et al., 2009). Curcumin also inhibits the IP3 receptor in the brain (Dyer et al., 2002). Although recent evidence suggests that curcumin stimulates an intracellular Ca^2+^concentration increase in C2C12 myoblast, the effects of curcumin on skeletal muscle calcium channels remain unexplored (Wang et al., 2024).

Resveratrol is a polyphenol molecule found in grapevines and grape juice. Resveratrol has beneficial effects on health and anti-aging properties (Liao et al., 2017; Yu et al., 2024; Muhammad and Allam, 2018). Resveratrol stimulates skeletal muscle regeneration and maintenance in aging (Munguía et al., 2022). Interestingly, resveratrol modulates the alternative splicing of exon 83 of RYR1 by reducing the level of CUG-BP1 (also called CELF1), an RNA-binding protein regulating alternative splicing and mRNA stability (Kajdasz et al., 2022). Resveratrol stimulates the inclusion of RYR1 exon 83, which is known to be lacking in Myotonic Dystrophy type I (DM1) (Santoro et al., 2020). In rat ventricular myocytes, a derivate of resveratrol called polydatin inhibits the cardiac L-type voltage-gated Ca^2+^ channels (Karami et al., 2022). Polydatin increases the frequency of calcium sparks, suggesting a positive modulation of RYR. Inhibition of the nitric oxide synthase prevented the action of Polydatin on both channels (Deng et al., 2012). Catechins extracted from green tea leaves directly increase the activity of RYR1, and in flexor digitorum brevis fibers significantly potentiate the calcium transient evoked by tetanic electric pulse stimulation (Feng et al., 2010).

Recently, oleuropein, a phenolic compound from olives was identified in a screening of 5000 bioactive molecules to stimulate an increase in mitochondrial Ca^2+^ concentration (Gherardi et al., 2025). Oleuropein binds the MCU regulator one protein, activating the MCU that in turn increases the Ca^2+^ uptake in mitochondria. The mitochondria energy metabolism and muscle performance are enhanced. Oleuropein reversed the age- and sarcopenia-induced decrease in mitochondria Ca^2+^ uptake in human myotubes. Chronic dietary of oleuropein enhanced mitochondrial function in aged rats and restored their physical performance (Gherardi et al., 2025). The 1,4 benzothiazepine derivate S107 molecule prevents the dissociation of RYR1 and FKBP12 (calstabin-1), stabilizing the RYR1 in its closed conformation, thus reducing the RYR1-induced Ca^2+^ leak from SR occurring in skeletal muscle from the elderly (Andersson et al., 2011).

#### Improving mass muscle in older adults

Clinical trials have tested the combination of natural bioactive compounds with endurance or resistance physical training in older adults (Alway et al., 2017). A trial of endurance training combined with tea catechins supplementation in women over 75 with sarcopenia conditions shows a significant improvement in leg muscle mass and walking speed (Kim et al., 2013). Another trial combining tea catechins with resistance training and essential amino acids improves skeletal muscle mass (Tokuda and Mori, 2023).

Rycal S48168 (ARM210), a molecule that restores the RYR1-clastabin1 association, can prevent the SR Ca^2+^ leak and cure RYR1-related myopathies (Todd et al., 2022). The phase-one study of Rycal S48168 was recently published (Todd et al., 2024). The potential of Rycal S48168 on muscle atrophy and sarcopenia remains to be tested.

Mitochondrial transplantation has emerged as a promising therapy to help cure patients with various pathologies such as obesity, diabetes, neurodegenerative diseases, and heart ischemia (Kim et al., 2023). MT can also potentially improve muscle regeneration following injury and in aging patients to mitigate sarcopenia (Alway et al., 2023; Tian et al., 2023b). A recent study showed that MT recovered the impaired Ca^2+^ homeostasis in hypertrophic cardiomyocytes (Li et al., 2024). The potential of MT in restoring the Ca^2+^ homeostasis in aging skeletal remains to be explored.

## Conclusion and future perspectives

Aging affects the expression, function, interaction, and localization of Ca^2+^ channels in skeletal muscles. Altering Ca^2+^ channels’ function and expression impairs the Ca^2+^-dependent intracellular signaling in skeletal muscles. What could be the onset of Ca^2+^ channels altered expression when skeletal muscles are aging? In aging, the lack of physical activity leads to the reduction of skeletal muscle mass and, therefore, protein expression. The absence of skeletal muscles’ mechanosensors stimulation might modify the expression of Ca^2+^ channels or Ca^2+^ channel regulatory proteins (Vanmunster et al., 2022). The consequences are muscle mass loss, reduced regeneration capacity, and physical performance of elderly people. Muscle mechanical stimulation with regular physical activity, nutrition habits, and bioactive molecules can help preserve skeletal muscle mass and performance while aging. Different channels generate Ca^2+^ signals essential to stimulate myogenesis, TRPC, Orai1, Piezo1. How do these channels coordinate their activities? What signals regulate their activities? And what are the specific downstream signals activated by Ca^2+^ passing through each channel? Their exact contributions in the different stages of myogenesis remain unclear. Particularly, Piezo1 expression and activity in aging remain to be explored. Piezo1, as a mechanosensing channel, might play a role in the onset of aging muscle atrophy and sarcopenia. The molecular mechanisms of the beneficial effects of bioactive molecules and exercise on Ca^2+^ channels are starting to be investigated, and results will help, in the future, to preserve healthy skeletal muscles in elderly people.

## Glossary

ECC: Excitation-Contraction Coupling
ER/SR: Endoplasmic/Sarcoplasmic Reticulum
RYR1: Ryanodine Receptor Type 1
DHRP: Dihydropyridine Receptors
VGCCs: Voltage-gated Ca^2+^ Channels
LVA: Low Voltage Activation
HVA: High Voltage Activation
IGF-1: Insulin Growth Factor-1
CREB: cAMP Response Element Binding Protein
TnT-T3: Troponin T3
CRU: Ca^2+^ Release Unit
JPs: Junctophilins
FKBP-12: Calstabin-1
ATP11A: P-type Adenosine Triphosphatase
KLF15: Krueppel-like Factor 15
IL-6: Interleukin-6
SOCE: Store-Operated Calcium Entry
STIM: Stromal Interaction Molecule
SERCA: Sarcoplasmic-Endoplasmic Reticulum Calcium ATPase
DMD: Duchenne Muscular Dystrophy
TRP: channel Transient Receptor Potential Channel
CaN: Calcineurin
MEF2: Myocyte Enhancer Factor-2
SOAR: STIM1 Orai-activating Region
CID: Combined Immunodeficiency
TAM: Tubular Aggregate Myopathy
IP3R: Inositol 1,4,5-trisphosphate Receptor
GPCR: G-protein-coupled Receptor
PLC: Phospholipase C
IP3: Inositol 1,4,5-trisphosphate
DAG: Diacylglycerol
PKC: Protein Kinase C
NMJ: Neuromuscular Junction
AChR: Acetylcholine Receptor
SCS: Slow Channel Myasthenic Syndrome
CICR: Calcium-induced Calcium Release
PASMC: Pulmonary Arterial Smooth Muscle Cells
ANO1: Ca^2+^-activated Chloride Channel
MAMs: Mitochondria-associated Membranes
MH: Malignant Hyperthermia
MCU: Mitochondrial Ca^2+^ Uniporter
Mfn2: Mitofusin 2
CCD: Central Core Disease
ROS: Reactive Oxygen Species
RNS: Reactive Nitrogen Species
MICU: Mitochondrial Calcium Uptake Family Member
PV: Parvalbumin
Mcub: Mitochondrial calcium uniporter dominant-negative subunit beta
PDK4: Pyruvate Dehydrogenase Kinase 4
CsA: Cyclosporine A
CuZnSOD: CuZn-superoxide Dismutase
FDB: Flexor Digitorum Brevis
DM1: Myotonic Dystrophy type I
